# Neurodiversity and Artistic Performance Characteristic of Children With Autism Spectrum Disorder

**DOI:** 10.3389/fpsyg.2018.02594

**Published:** 2018-12-18

**Authors:** Nobuo Masataka

**Affiliations:** Primate Research Institute, Kyoto University, Kyoto, Japan

**Keywords:** children’s drawing, autism spectrum disorder, neurodiversity, giftedness, folk psychology

## Abstract

Neurodiversity refers to the notion that seemingly ‘impaired’ cognitive as well as emotional features characteristic of developmental disorders such as autism spectrum disorder (ASD) fall into normal human behavioral variations that should enjoy some selective advantages. In the present experiment, the author compared what was depicted in subjects’ drawings after they experienced an identical event, e.g., going on a picnic to a garden in the vicinity of their nursery school, between children with ASD and IQ-matched, typically developing (TD) children. When the material was coded according to types of drawn objects, such as human, animal, plant, food, vehicle, building, and others, the overall variability of the objects did not differ between TD children and children with ASD. However, TD children were more likely than children with ASD to depict human images. Conversely, other objects were more likely to be drawn by children with ASD than by TD children. While TD children were more likely to focus on humans than on non-human objects when drawing, children with ASD were more likely to focus on non-human objects than on humans even after both had experienced an identical event. The author argues that such findings are empirical evidence for the claim that there is some selective advantage of enhanced capabilities characteristic of ASD, i.e., neurodiversity, that may represent a balance toward “folk physics” at the expense of “folk psychology.”

## Introduction

So far, autism has been regarded as a spectrum of “developmental disorders that are characterized by social communicative difficulties and restricted behaviors and interests” (American Psychiatric Association, 2013). Concerning negative attributes in association with social context, in particular, individuals with autism spectrum disorder (ASD) are considered to have marked difficulties in social interactions such as “making eye contact, engaging in reciprocal interactions, and responding to emotional cues of others” (Dawson et al., 2005). “Basic impairments, such as lack of attention to others, often appear within the first year of life” (Werner et al., 2000). Infants who are later diagnosed with ASD exhibit a mean decline in duration of visual fixation from 2 to 6 months of age (Dawson et al., 1998). From 2 to 3 years of age, impairments come to be recognized extensively with respect to social orientation such as joint attention, imitation, response to others’ emotional expressions, and face recognition (Sigman et al., 1992; Dawson et al., 2002, 2004). “Many of the early social impairments in ASD involve the ability to attend to and process information from faces” (Masataka, 2017a). This has researchers led researchers to reason that, “in ASD, impairments in face and emotion processing might play a fundamental role in the dysfunction of the neurological mechanism” (Masataka, 2017a) that underlies the impairments with regard to social cognition (Dawson et al., 2005).

While autism has been regarded as a disorder, some researchers have recently come to see it more in the light of a ‘strength-oriented view’ of ASD in terms of the utility of ASD in neurodiversity (Masataka, 2017a). According to a report published in 2012, ASD affects approximately 1 in every 88 individuals in the United States, and is considered to be congenital, lifelong, and extremely heritable (Constantino et al., 2013). Accumulated evidence suggests that “as many as 300 to 500 distinct genes are involved in the etiology, with no single locus accounting for more than 1% of cases” (State and Sestan, 2012). As its consequence, ASD is believed to be a diagnosis made purely on the basis of behavior (Lord et al., 2011). The genetic heterogeneity of ASD poses a great challenge for understanding the condition biologically: “with so many different ‘causes,’ a key question is how this genetic heterogeneity can be instantiated into common forms of this disorder” (Jones and Klim, 2013).

In order to address this question, some selective advantages have been claimed for ASD, such that ASD may represent a balance toward “folk physics” at the expense of “folk psychology” (see Spikins et al., 2016 for review). These enhanced abilities may extend beyond technical skills and include heightened sensory sensitivities. In fact, Spikins et al. (2017) reported the results of an analysis of the relationship between the autism quotient and attitudes toward valued personal possessions and found that “individuals with ASD show a reduced tendency to value and preserve objects as reminders of relationship/attachment figures and place a greater value on the direct practical function of their personal possessions.” They argued that “the latter strategy may have been more selectively advantageous in certain contexts whereas it was less advantageous in others in the distant evolutionary past.” Here the author presents data collected according to the identical experimental paradigm as that adopted by Spikins et al. (2017), focusing on the issue of drawing performed by children with ASD as well as by typically developing (TD) children.

Concerning artistic performance shown by children with ASD, a unique phenomenon is well-known anecdotally. Typically, landscapes, including animals, can be depicted (and presumably perceived) with exceptional realism, whereas the complete human figure, including the head, of a human being as a social being is ignored, or, if not ignored, can be only crudely depicted. Grandin (1996) and Grandin and Cook (2004) has called such a unique manner of perception of the landscape (or, using her expression, the “brain’s representation of the world”) “thinking in pictures.” Being a person with ASD, she describes her own acute visual processes and her particular ability to employ visualization to concretize events and concepts (an outstanding characteristic of many individuals with ASD), and states that “one of the most profound mysteries of autism has been the remarkable ability of most autistic people to excel at visual spatial skills.” When she was a child, she thought “everyone thought in pictures.” Her descriptions of her thought processes, such as her description “when I do an equipment simulation in my imagination or work on an engineering problem, it is like seeing a videotape in my mind,” are instructive in light of the striking realism with which landscapes are depicted by individuals with ASD. In order to create new images, she takes “many little parts of images” she has “in the video library” of her imagination and pieces them together. She describes that she has video memories of every item she has ever worked with. Most significantly, Grandin (1996) reports that personal relationships themselves “made absolutely no sense to me until I developed visual symbols of doors and windows” with which to visualize the give and take of social interaction.

It seems reasonable to assume that the characteristics of visual memory and ability to “think in pictures” possessed by Grandin could be shared somehow by most individuals who are diagnosed as having ASD. Moreover, given the fact that autism is for the most part highly heritable and subject to some elements of positive selection, these capabilities might be advantageous within the perspective of neurodiversity. Nevertheless, no study of such possible advantage(s) has been attempted systematically so far. This study is a first step to pursue this issue. Here, the author quantitatively compared what children with ASD and TD children drew after experiencing an almost identical event, on the basis of the hypothesis that individuals with ASD would be more likely to focus on non-human objects than on human figures as the subject of their drawing activity.

Here, the author reports the results of an analysis of what was depicted in children’s drawings after they experienced an identical event, e.g., going on a picnic to a garden in the vicinity of their nursery school, as a function of their scores in a well-validated autism spectrum questionnaire, the autism quotient (AQ) (Auyeung et al., 2008). This is a parental-report measure designed to assess traits on the autism spectrum, extensively used and showing a large difference between the means of children who are TD and those with ASD. The general consensus is that the value 76 is the cut-off score for making a diagnosis of ASD. Following this criterion, in the present experiment, the author collected data from children diagnosed as ASD according to DSM-5 criteria (American Psychiatric Association, 2013) who exceeded this cut-off score in AQ as well as from TD children who were not found to exceed the score. Since ASD is diagnosed on the basis of behavior that is individually variable, such variability reflected in AQ variance would somehow relate to the diversity of drawing pattern shown by the participants. In order to examine this possibility, therefore in the present study, each of the groups of the children with ASD and of the TD children was divided into different two groups, further, one containing such children with relatively higher AQ scores and the other containing such children with relatively lower AQ scores.

## Materials and Methods

### Ethics Statement

This investigation was conducted according to the principles expressed in the Declaration of Helsinki. All experimental protocols were consistent with the Guide for Experimentation with Humans, and were approved by the Institutional Ethics Committee, of the Primate Research Institute, Kyoto University (#2016-150). The authors obtained written informed consent from the parents of all child participants, involved in the study.

### Participants

As outlined above, a total of four groups of 5- to 6-year-old children participated: two groups of 28 children (20 boys and 8 girls) who had been diagnosed as meeting DSM-5 diagnostic criteria for ASD (American Psychiatric Association, 2013) without any anxiety or phobic symptom by psychiatrists from several hospitals in Osaka Prefecture, Japan, and two groups of 28 TD children (20 boys and 8 girls) in Japan. All of these children went to a nursery school. At the commencement of the study, all of them were assessed using AQ (Auyeung et al., 2008). According to the results of the measurement, the children with ASD were subcategorized into two groups: one group in which the AQ score exceeded 105, and another group in which the score of the child was between 76 and 105 (76 being the cut-off score for making a diagnosis of ASD). Similarly, the two groups of TD children were categorized so that the AQ score of children in one group was between 36 and 76, while the score of children included in the other group was less than 36.

The cognitive ability of each participant was assessed using WISC-IV. Scores of Full-Scale IQ (Verbal IQ; Performance IQ) were 101.6 (99.1; 100.5) and 100.9 (98.7; 100.2) for the group of children with ASD with AQ score greater than 105, and the group with AQ between 76 and 105, respectively, whereas the scores were 103.0 (102.5; 100.1) and 100.6 (99.2; 100.3) for the group of TD children with AQ score between 36 and 75, and that with AQ score smaller than 36, respectively. The scores did not differ significantly with respect to the four groups overall (*F*_3,108_ = 0.41, *p* = 0.54, $\eta_{p}^{2}$ = 0.046 for Full-Scale IQ, *F*_3,108_ = 0.37, *p* = 0.69, $\eta_{p}^{2}$ = 0.038 for Verbal IQ and *F_3,108_* = 0.43, *p* = 0.50, $\eta_{p}^{2}$ = 0.029 for Performance IQ).

### Procedure

Each participant experienced an event of a ‘picnic’ organized by the nursery school on either of five weekdays with fine weather in the spring season between 2016 and 2017. In the event, the children, who belonged to subgroups consisting of 8- to 13 children, experienced roughly 1-h walk to a large park in the vicinity of the school and stayed there for another 1 h. During that time, they ate a lunch that had been prepared for them, and thereafter they went back to the school.

After returning to the school and taking a short break, each of the children was notified that “Now is the time for drawing (“Oekaki no jikan” in Japanese).” Each of the children was presented with a piece of 210 mm × 300 mm white bond paper, and was instructed to draw about the ‘picnic’ using their choice of 24 different colored pencils; “You can draw with these pencils what was most impressive in your experience of the today’s picnic.” The drawing period lasted for 30 min, and, then the drawing was collected by teachers of the school and was submitted for the following analysis.

For the analysis, types of objects depicted in each drawing were coded using categories, which were as follows: human, animal, plant, food, vehicle, building, and others. The author’s own preliminary study that had been conducted prior to the present one had revealed that these categories were almost exhaustive for coding of what was drawn about such a ‘picnic’ by children. Coding was undertaken by two coders, independently. Actually, they were nursery school teachers with no previous experience as coders. However, they had abundant knowledge with children’s drawings in general. They had not been notified about the purpose of the study, but were just told that the children’s drawings were of interest. When the results of the coding were inconsistent between the coders. they discussed the inconsistency, and one of the two altered her judgment to match that of the other. Actually, such an incident took place only twice during the study. The coding was conducted as a given object being ‘present (depicted)’ or ‘absent (not depicted)’ in each drawing, so that the objects each child had drawn were categorized by a one-zero sampling method. As a result, comparisons of the number of children who had depicted each categorized object in their drawing was possible among the four participant groups.

## Results

**FIGURE 1:**
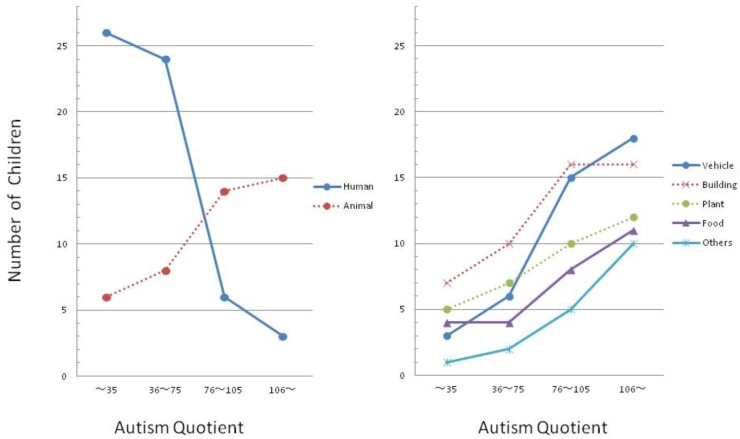
Comparisons of objects children chose as the subject of their drawing as a function of autism quotient (AQ) (the participants included in two groups whose AQ scores exceeded 75 were children diagnosed with autism spectrum disorder (ASD) according to DSM-5 while the participants included in the other two groups were TD children).

The results of the experiment are presented in Figure 1, which shows the number of children who depicted each of the seven categorized objects in the four participant groups. Since the present experiment was correlational in design, including such children varying in AQ scores as noted, the collected data were analyzed by a 7 (type of drawn object, OBJECT) × 4 (participant group that was classified according to AQ score, PARTICIPANT) analysis of variance. Results of the analysis revealed that the main effect was not statistically significant for either OBJECT (*F*_6,21_ = 0.85, *p* = 0.14, $\eta_{p}^{2}$ = 0.026) or PARTICIPANT (*F*_3,24_ = 0.32, *p* = 0.36, $\eta_{p}^{2}$ = 0.033). However, the interaction between these two factors was significant (*F*_18,9_ = 3.95, *p* = 0.014, $\eta_{p}^{2}$ = 0.497).

*Post hoc* pair-wise comparisons (McNemar tests) of scores between all possible pairs of the four participant groups with regard to each of the seven objects revealed that with regard to human figures, the number of TD children (whether their AQ scores were smaller than 36, or between 75 and 36) who had drawn the figure significantly exceeded the number of children with ASD (whether their AQ scores were greater than 105, or between 105 and 76) who had drawn it (*p*s < 0.05). With regard to the other six objects, conversely, the number of children with ASD (whether their AQ scores were greater than 105, or between 105 and 76) who had drawn the object significantly exceeded the number of TD children (whether their AQ scores were smaller than 36, or between 36 and 75) who had drawn it (*p*s < 0.05).

## Discussion

When the material depicted in drawings was coded according to the type of drawn objects, such as human, animal, plant, food, vehicle, building, and others, the overall variability of the objects depicted did not differ between TD children and children with ASD. Nevertheless, TD children were more likely than children with ASD to depict human images. Conversely, others objects were more likely to be drawn by children with ASD than by TD children. The results clearly confirm the author’s hypothesis, revealing the fact that while TD children are more likely to focus on humans than on non-human objects, children with ASD are more likely to focus on non-human objects than on humans, even after both had experienced an identical event.

In this regard, it should be noted that while a lot of attention concerning ASD focuses on negative behaviors such as those described above in the social context, a number of positive attributes associated with ASD have also been revealed in the non-social context. Individuals with this disorder are likely to be “particularly skilled at perceiving details as opposed to whole gestalts. Children with ASD perform better than TD children on the block design test of the Wechsler Intelligence Scale for Children (WISC-IV), which requires taking blocks that are all white, all red, or a mixture of red and white, and putting them together to match a preexisting pattern” (Baron-Cohen, 2003). They also perform better than TD children on tests of detection of patterns embedded in more complex patterns and often exhibit superior drawing techniques compared to TD children (Karolyi et al., 2003), though such findings have, so far, led some researchers to suggest that individuals with ASD experience what has been termed “weak central coherence,” namely, “they fail to grasp the whole of a situation and perceive mainly the constituent parts (Armstrong, 2012),” a deficit-oriented view of the disorder.

As far as TD children concerned, the fact is known that boys are more likely to be interested in machines and vehicles and to depict such objects under free drawing situation than girls, who are more likely to be interested in human figures and dolls and to depict them than boys (Bell et al., 1981). However, boy-like drawing pattern is observed in girls with congenital adrenal hyperplasia in which condition excessive level of androgen is secreted (Iijima et al., 2001). The linkage among organizational effect of this hormone, the presumed cause of hyper-masculinization of the brain, and atypical pattern of drawing activity has been reported by more recent studies (Turgeon, 2008; Doi and Shinohara, 2018). Such argument might remind one of the hyper-male brain theory of autism (Baron-Cohen, 2003).

Taken together, the present findings would indicate the possibility that the difference of the drawing found between the TD children and the children with ASD was due to the hyper-masculinity generated by ASD, and that such characteristics could have been more advantageous selectively in the evolutionary past. This reasoning is also consistent with the conclusion drawn from the recent review of evidence about the similarities between characteristics of the Ice Age drawings and paintings discovered in the Lascaux and Chauvet caves and drawings and paintings produced by individuals with ASD (Masataka, 2017a,b). While animals’ images have been found to be produced abundantly in both of these samples, human images are extremely rare. Moreover, both are extremely realistic when they depict animals, whereas human figures may be only crudely depicted. This might be because the animal images in the cave art were produced via visual realism by their creators, but the human figures were produced based on the creators’ own knowledge of humans (via intelligence-based realism), as are images produced by contemporary individuals with ASD, who have impaired cognition of interpersonal communication. This represents a significant revision of a similar proposal from 20 years ago that now takes into account the acquisition of new knowledge and understanding of ASD. The author places this disorder more in the light of a ‘strength-oriented view’ of ASD in terms of the utility of ASD in neurodiversity, which had survival advantages, especially for hunter-gatherer groups.

“Neurodiversity refers to the notion that seemingly ‘impaired’ cognitive as well as emotional properties characteristic of developmental disorders such as ASD, a neurodevelopmental disorder with unusual sensory processing, are not necessarily deficits, but fall into normal behavioral variations exhibited by humans. Stated more formally, this notion was recently described as “a concept where neurological differences are to be recognized and respected as any other human variation” (Armstrong, 2012). The term neurodiversity was first coined in the late 1990s by New York journalist Harvey Blume and Australian autism activist Judy Singer, and has become an important component of the civil rights movement for those with neurologically based disabilities. While neurodiversity is indeed a paradigm shift from a “deficit-oriented view of ASD” to a “strength-oriented view of ASD” (Silberman, 2015), experimental and empirical scientific evidence confirming this conceptual notion and paradigm has been meager” (Masataka, 2017b). Certainly the present analysis still remains preliminary. Further analysis such as comparison of the usage of color as well as of perspective is required between TD children and children with ASD, which should be a next step of the future research. Nonetheless, the present quantitative findings apparently support ‘a neurodiversity hypothesis’ to explain a unique characteristic of individuals with ASD regarding memory about visual experience and its expression.

## Author Contributions

NM designed the study, collected and analyzed the data, and drafted the manuscript.

## Conflict of Interest Statement

The author declares that the research was conducted in the absence of any commercial or financial relationships that could be construed as a potential conflict of interest.
